# α-Cyperone Confers Antidepressant-Like Effects in Mice *via* Neuroplasticity Enhancement by SIRT3/ROS Mediated NLRP3 Inflammasome Deactivation

**DOI:** 10.3389/fphar.2020.577062

**Published:** 2020-10-08

**Authors:** Baomei Xia, Yue Tong, Changbo Xia, Chang Chen, Xin Shan

**Affiliations:** ^1^Faculty of Rehabilitation Science, Nanjing Normal University of Special Education, Nanjing, China; ^2^Department of Basic Medical Sciences, University of Arizona College of Medicine—Phoenix, Phoenix, AZ, United States; ^3^School of Basic Biomedical Science, Nanjing University of Chinese Medicine, Taizhou, China; ^4^Department of Neurology, Nanjing Hospital of Chinese Medicine Affiliated to Nanjing University of Chinese Medicine, Nanjing, China; ^5^Hanlin College, Nanjing University of Chinese Medicine, Taizhou, China

**Keywords:** depression, SIRT3, ROS, NLRP3 inflammasome, neuroplasticity, α-cyperone

## Abstract

α-Cyperone (Cy) is a major active compound of Cyperus rotundus that has various pharmacological activities. But whether Cy possesses antidepressant effect is unknown. In this study, we exposed mice to chronic unpredictable mild stress (CUMS) with or without intervention with Cy. Our results showed that Cy significantly improved the depressive phenotypes in sucrose preference test, tail suspension test and forced swimming test. Meanwhile, increased SIRT3 expression, reduced ROS production and activated NF-κB signal were detected in the hippocampus of mice. NLRP3 inflammasome related proteins including NLRP3, ASC, Caspase-1, IL-1β, IL-18 and GSDMD-N were downregulated after Cy administration. Synaptic proteins including Synapsin-1 and PSD-95 and dendritic spine density were improved after Cy treatment. Moreover, the protective effects of Cy in CUMS mice were compromised when co-administrated with SIRT3 inhibitor 3-TYP. Taken together, these findings suggested that Cy has therapeutic potential for treating depression and that this antidepressant effect may be attributed to SIRT3 stimulated neuroplasticity enhancement by suppressing NLRP3 inflammasome.

## Introduction

Depression is a psychiatric disorder which affects more than one-fifth of the global population (Wang et al., 2019). It causes considerable burden on individuals and society with its high morbidity, recurrence, and mortality (Feng et al., 2019). Currently, a number of antidepressants including mainstream antidepressants SSRIs are used in the clinical treatment of depression. However, disadvantages such as delayed onset time, inadequate response rate, and side effects such as sexual dysfunction, sleep disorders, and suicidal tendencies limited the clinical usages of those drugs (Clayton et al., 2018). Therefore, the development of novel antidepressants with high efficacy and safety is urgently needed.

Neuroplasticity is the ability of the brain to adapt to new conditions, including synapse turnover, dendritic remodeling, neurogenesis, and long-term potentiation (LTP) (Laura et al., 2018). Compelling evidence indicates that synaptic plasticity is implicated in the development of depression. Microarray gene profiling revealed the reduction of synapse-related genes and loss of synapses in hippocampus and prefrontal cortex from subjects diagnosed with MDD (Menard et al., 2016). Therapies exerted rapid and significant antidepressant effects *via* dendritic spine remodeling and neuroplasticity related protein enhancement (McAvoy et al., 2015; Menard et al., 2016).

Neuroplasticity can be affected by various signals, among which is the NLRP3 inflammasome (Qi et al., 2018). Synaptic failure was observed in mice with NLRP3 inflammasome activation, and blockage of NLRP3 inflammasome with repeated Mcc950 treatment exerted beneficial effects on neuroplasticity, such as long-term potentiation (Qi et al., 2018). The NLRP3 inflammasome is comprised of the NLRP3 protein, adapter protein ASC, and Caspase-1. It plays a pivotal role in innate immunity by stimulating Caspase-1. Activated Caspase-1 promotes the secretion of IL-1β, IL-18 and GSDMD, consequently leading to the impairments of neuroplasticity (Qi et al., 2018; Shao et al., 2018). ROS is a crucial activator in triggering NLRP3 inflammasome activation directly or indirectly by acting on NF-κB (Teng et al., 2020). SIRT3, a NAD+-dependent deacetylase that mainly located in mitochondria, modulates signal pathways to control ROS generation (Teng et al., 2020). SIRT3 maintains the ROS homeostasis by targeting mitochondrial enzymes such as superoxide dismutase 2 (SOD2), which transforms harmful superoxide radicals into nontoxic oxygen or hydrogen peroxide (Zheng et al., 2018).

α-Cyperone is a major active compound of Cyperus rotundus. It has been reported to possess a variety of pharmacological activities. Azam Azimi et al. found that α-Cyperone has a pronounced effect on the destabilization of microtubule fibers in brain *via* interacting with tublin (Azimi et al., 2016). A study using LPS-treated RAW 264.7 macrophages observed significant anti-inflammatory actions after α-Cyperone intervention, which was related to NF-κB induced reduction of COX-2 and IL-6 expression (Jung et al., 2013). LIU and his colleagues explored the effect of Cy on lung injury, and discovered that Cy could profoundly protected mice from lung injury, which was tightly linked to the suppression of NLRP3 and NF-κB signaling (Liu et al., 2019). Nevertheless, little is known about the antidepressant property of Cy. Hence, the aim of this study was to investigate the antidepressant activity of Cy and its potential mechanism.

We performed behavioral tests in CUMS-challenged mice to evaluate the antidepressant action of Cy. The SIRT3/ROS/NF-κB signaling, NLRP3 inflammasome activation and neuroplasticity in terms of synaptic proteins and dendritic spine density were also observed. Finally, the antidepressant effects of Cy in a condition of SIRT3 inhibition was determined.

## Materials and Methods

### Animals

Male adult C57BL/6 mice were used in this study. Mice were housed under a constant temperature of 23°C with a 12-h light-dark cycle. Animals were provided with food and water *ad libitum* and allowed to habituate to the novel environment for 1 week. All animal care procedures were approved by the Institutional Animal Care and Use Committee at Nanjing University of Chinese medicine, and performed in accordance with the Provision and General Recommendation of Chinese Experimental Animals Administration Legislation.

### Drugs

Cy (98% purity) was provided by Aoke Biology Research Co., Ltd (Beijing, China). Fluoxetine (Flu) was obtained from Sigma-Aldrich (St. Louis, USA). 3-TYP was purchased from MedKoo Biosciences, Inc (Chapel Hill, USA). Both α-cyperone (0.5 mg/ml; 1 mg/ml) and Fluoxetine (2 mg/ml) were dissolved in 0.05% DMSO and intragastrically administrated at a volume of 10 ml/kg.

### Experimental Design

#### Experiment I: Effects of Cy in CUMS Mice

Mice were randomly divided to five different groups (n=12 per group): control, CUMS, CUMS + Cy (5mg/kg), CUMS + Cy (10mg/kg), CUMS + Flu (20mg/kg). After 1 week of adaption, mice in the control group were housed in normal conditions and mice in other groups were subjected to chronic unpredictable mild stress for 5 weeks. Drugs were daily (8:00 am) intragastrically administered the last 2 weeks (Week 4–5) of the CUMS protocol, and then behavioral assessments including SPT (Day 36), OFT (Day 37), TST (Day 37), and FST (Day 38) were performed in sequence. At the end of the experiment (Day 38), mice were anesthetized with 1% sodium pentobarbital. Brains were rapidly removed and hippocampi were dissected on a cold ice and stored at −80°C for further analysis.

#### Experiment II: Influence of SIRT3 Inhibition in Cy-Related Effects in CUMS Mice

Animals were randomly divided to five different groups (n=12 per group): control, CUMS, CUMS + Cy (10 mg/kg), CUMS +3-TYP (10 mg/kg), CUMS + Cy (10 mg/kg) +3-TYP. After 1 week of adaption, mice in the control group were housed in normal conditions and mice in other groups were subjected to chronic unpredictable mild stress for 5 weeks. Cy were daily (8:00 am) given the last 2 weeks (Week 4–5) of the CUMS protocol, and 3-TYP were injected 1 h prior to Cy administration. After drug administration phase, behavioral assessments including SPT (Day 36) and FST (Day 37) were performed in sequence. At the end of the experiment (Day 37), mice were anesthetized with 1% sodium pentobarbital. Brains were rapidly removed and the hippocampi were dissected on a cold ice and stored at −80°C for further analysis.

### CUMS Procedure

Mice were subjected to various chronic unpredictable mild stress for 5 weeks, including wet bedding for 12 h, no bedding for 12h, cage shaking on a 120 rpm rocking bed for 40 min, 45°cage tilt for 12 h, restraint in a 50 ml centrifuge tube (in a 50 ml conical tube (several ventilation holes for breathing) for 6h, food deprivation for 12 h and water deprivation for 12 h. Animals were exposed to 1 or 2 of the stressors each day on a randomized schedule. Control animals were handled only for necessary procedures such as routine cage cleaning.

### Behavioral Tests

#### Sucrose Preference Test

SPT was used to determine the anhedonia state of animals. Each animal was trained to adapt to 2% sucrose solution (w/v) by exposure to two bottles of sucrose solution for 3 days. Then mice were deprived of water for 18 h, after which each mouse was allowed to consume from both bottles for 2 h, one containing tap water and the other containing 2% sucrose solution. To avoid side preferences, the positions of the two bottles were switched in the middle of the test. The sucrose preference index was calculated using the following formula: (sucrose intake/total fluid intake) × 100%. n=12 per group.

#### Tail Suspension Test

Each mouse was suspended with tape 1 cm from the tip of its tail. Animals were suspended for 6 min, and the duration of immobility during the final 4 min was recorded. Mice were considered immobile when they hung passively and were completely motionless. n=12 per group.

#### Force Swimming Test

Briefly, mice were individually placed and forced to swim in a glass cylinders (height, 30 cm; diameter, 16 cm) containing 25 cm of water, maintained at 24 ± 1°C. The test lasted for 6 min, and the immobility time during the last 4 min of the test was recorded. A mouse was judged to be immobile when it remained floating passively or immobile in the water. n=12 per group.

#### Open Field Test

The locomotor activity of the mice was evaluated applying a mouse spontaneous activity video analysis system (Shanghai Mobile Datum Information Technology Company, Shanghai, China). Mice were placed in the corner of the open field apparatus (50 cm × 50 cm × 40 cm). After 5 min, total distance traveled and time spent in the central area (25 cm × 25 cm) were automatically analyzed by the mouse spontaneous activity video analysis system. The experimental apparatus was cleaned with 70% ethanol after each test. n=12 per group.

### Reactive Oxygen Species Measurement

ROS level assay was carried out using Dihydroethidium (DHE, SIGMA D7008) fluorescence as previously reported (Gentile et al., 2018). The brain sections (300 μM) were incubated with 50 μM DHE in a light-protected area at room temperature for 60 min, then washed in PBS and fixed with 4% paraformaldehyde. ROS was observed under a fluorescent microscope. In the presence of superoxide, DHE undergoes oxidation and intercalates in cell DNA, thus staining the nucleus with red fluorescence (excitation at 525 nm, emission 610 nm). Images were analyzed using ImageJ. n=3 in Experiment I, n=4 in Experiment II.

### Western Blot

The hippocampus was weighed and homogenized in RIPA buffer containing protease inhibitors and phosphatase inhibitors. After the lysates were centrifuged at 12,000 g for 10 min at 4°C, supernatants were collected and protein concentrations were analyzed using bicinchoninic acid assay. Next, loading buffering 5X was added, and proteins were boiled for 5 min in a metal bath. Protein samples were run on SDS-PAGE, transferred onto polyvinylidene difluoride (PVDF) membranes, and then blocked with 3% BSA. The membranes were incubated with the following primary antibodies over night at 4°C: rabbit anti-SIRT3 (1:1000, CST), rabbit anti-p-NF-κB p65 (1:800, CST), rabbit anti-NF-κB p65 (1:1000, CST), rabbit anti-NLRP3 (1:800, Abcam), rabbit anti-ASC (1:1000, Abcam), rabbit anti-cleaved-Capspase-1 (1:1000, Abcam), rabbit anti-cleaved-IL-1β (1:1000, Abcam), rabbit anti-cleaved-IL-18 (1:1000, Abcam), rabbit anti-GSDMD-N (1:800, Abcam), rabbit anti-GSDMD (1:1000, Abcam), rabbit anti-PSD-95 (1:1000, CST), rabbit anti-Synapsin-1 (1:1000, CST), rabbit anti-GAPDH (1:1000, Abcam). HRP-conjugated Affinipure Goat Anti-Rabbit IgG (1:5000, proteintech) were incubated for 90 min at room temperature. The membranes were digitally scanned by an imaging system (Bio-Rad, USA) and then quantified using ImageJ software. The relative expression levels of all proteins were normalized to GAPDH. n=6 per group.

### Golgi Staining

Golgi staining was performed according to the instructions of the FD Rapid GolgiStain Kit (FD NeuroTechnologies, Ellicott City, MD). Briefly, Coronal brain sections (100 μm thickness) were cut on a freezing microtome. Slices were dehydrated in ethanol, cleared in xylene, and mounted in neutral balsam. Dendrites were imaged using a Leica SP2 confocal microscope.

The quantification for Golgi staining was conducted as previously described (Arroyo-Garcia et al., 2020). For dendritic spine density measurement, a length of a distal dendrite (termed distal after the 4th branching order) was traced (at least ≥10 μm long at 1000×), the exact length of the dendritic segment was analyzed, and the number of spines along the dendrite was calculated (to yield spines/10 µm). n=4 per group.

### Statistical Analysis

All values were expressed as mean ± standard error of the mean (SEM). Data were analyzed statistically using one-way analysis of variance (ANOVA), followed by *post hoc* analysis with Bonferroni multiple comparisons test. Statistical significance was defined as p < 0.05.

## Results

### Antidepressant Property of Cy in CUMS Mice

#### Effects of Cy on Depressive-Like Behaviors

**Figure 1:**
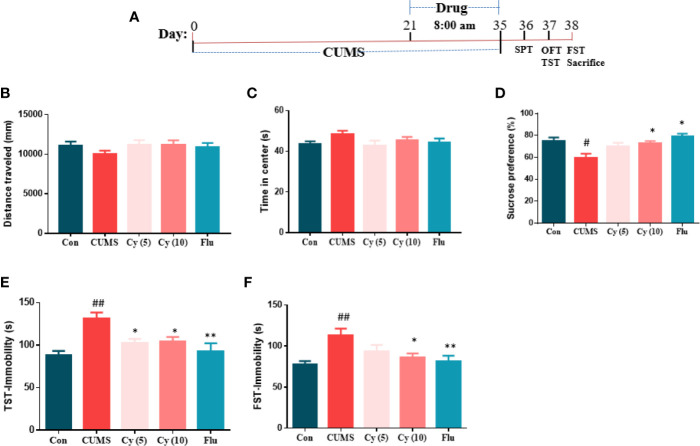
Experimental procedures and behavioral assessment after 5 weeks. Drug administration sustained 2 weeks during the CUMS procedure **(A)**. CUMS mice exhibited decreased sucrose preference rate in SPT **(D)**, and extended immobility time in TST **(E)** and FST **(F)**, which were all reversed by Cy treatment. No significant difference was found in OFT **(B, C)**. Data are represented as mean ± SEM. ^#^*p* < 0.05 and ^##^*p* < 0.01 vs. Con group. *p < 0.05 and **p < 0.01 vs. CUMS group.

After 2 weeks of drug administration, behaviors assessments were carried out (**Figure 1A**). In SPT assessment, the repeated CUMS exposure notably decreased the percentage of sucrose consumption as compared with control group (**Figure 1D**). Nonetheless, Cy (10 mg/kg) and Flu significantly increased the percentage of sucrose consumption.

In TST and FST studies, a significantly prolonged duration of immobility was observed in CUMS-challenged mice compared with controls (**Figures 1E, F**). Treatment with Cy (5 mg/kg, 10 mg/kg in TST; 10 mg/kg in FST) and Flu reduced the immobile time in CUMS mice in both tests, suggesting that Cy and Flu ameliorated the depression-like behaviors in mice induced by CUMS.

In OFT (**Figures 1B, C**), no significant among-group difference was discovered in total distance traveled or time spent in center, demonstrating that Cy and Flu improved depression-like behaviors induced by CUMS without affecting locomotor activity.

### Effects of Cy on SIRT3/ROS Signaling in the Hippocampus

**Figure 2:**
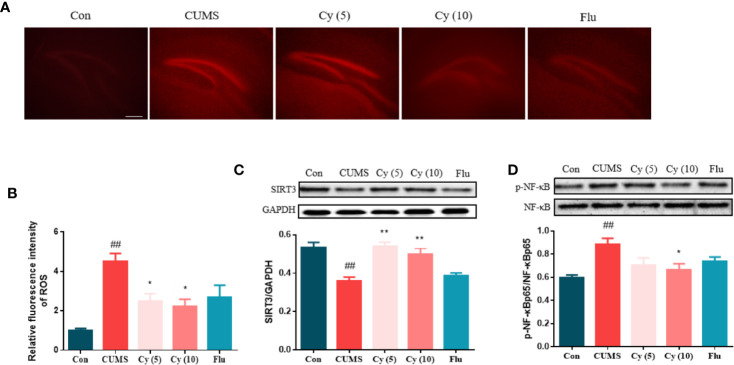
ROS production and protein levels of SIRT3 and NF-κB in the hippocampus. Hippocampal ROS production was increased in mice receiving CUMS challenge **(A, B)**. Meanwhile, upregulated SIRT3 expression **(C)** and downregulated p-NF-κB level **(D)** was detected in the hippocampus of CUMS mice. Nonetheless, the SIRT3/ROS/NF-κB signaling was successfully restored by Cy intervention. Data are represented as mean ± SEM. ^##^*p* < 0.01 vs. Con group. *p < 0.05 and **p < 0.01 vs. CUMS group.

DHE fluorescence staining showed that the ROS intensity was profoundly increased in CUMS group compared with control group (**Figures 2A, B**). However, after Cy intervention (5 mg/kg, 10 mg/kg), the ROS activity in CUMS mice was significantly inhibited, suggesting that Cy can prevent ROS formation in CUMS mice.

To investigate Cy’s effects on SIRT3 and NF-κB expression, Western blot was carried out. While CUMS modeling significantly decreased the SIRT3 protein level and increased p-NF-κB ratio, Cy treatment successfully (5 mg/kg, 10 mg/kg for SIRT3; 10 mg/kg for p-NF-κB) reversed p-NF-κB percentage and SIRT3 expression (**Figures 2C, D**).

#### Effects of Cy on Protein Levels of NLRP3 Inflammasome

**Figure 3:**
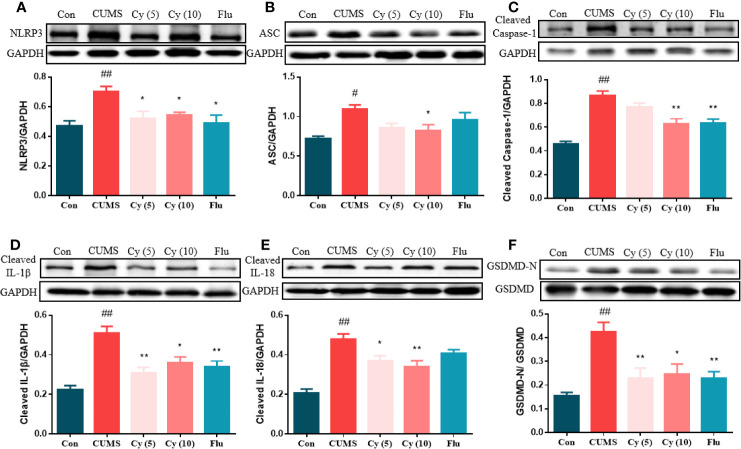
Protein levels of the NLRP3 inflammasome in the hippocampus. Western blot was performed to measure the protein levels of NLRP3 inflammasome. The expression of NLRP3 inflammasome complex NLRP3 **(A)**, ASC **(B)**, Caspase-1 **(C)** were elevated, and cleavage of IL-1β **(D)**, IL-18 **(E)**, and GSDMD **(F)** were activated after CUMS modeling. Cy treatment notably suppressed these expression. Data are represented as mean ± SEM. ^#^*p* < 0.05 and ^##^*p* < 0.01 vs. Con group. *p < 0.05 and **p < 0.01 vs. CUMS group.

To examine whether Cy could inhibit NLRP3 activation, we investigated the effect of Cy on NLRP3 inflammasome complex, the cleavage of IL-1β, IL-18, and GSDMD, which were strongly associated with the NLRP3 inflammasome activation. As shown in **Figure 3**, the protein expressions of NLRP3, ASC, cleaved Caspase-1, cleaved IL-1β, cleaved IL-18, and the percentage of GSDMD-N in the hippocampus of CUMS-treated mice were significantly upregulated as compared to those in Control group. Cy 5 mg/kg and Flu partially whereas Cy 10 mg/kg completely restored these proteins, indicating that Cy and Flu could inhibit NLPR3 inflammasome activation.

#### Effects of Cy on Neuroplasticity

**Figure 4:**
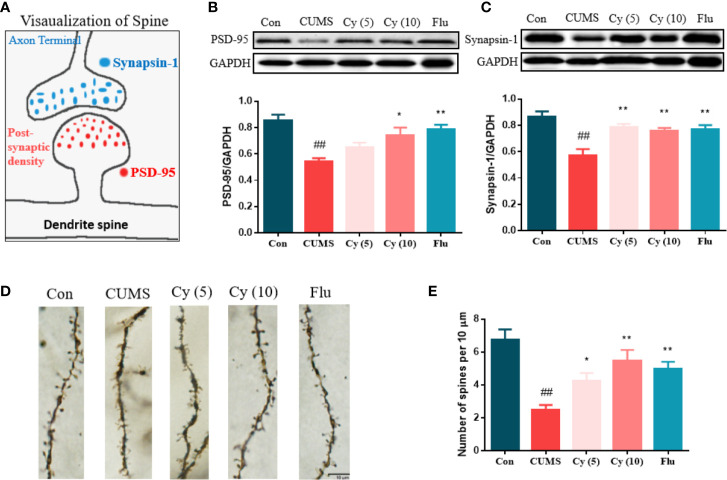
Synaptic proteins and dendritic spine density in the hippocampus. PSD-95 and Synapsin-1 are postsynaptic and presynaptic protein respectively **(A)**. Cy intervention significantly restored CUMS-induced decrease in PSD-95 **(B)** and Synapsin-1 **(C)**, as well as the deficiency in dendritic spine density **(D, E)**. Data are represented as mean ± SEM. ^##^*p* < 0.01 vs. Con group. *p < 0.05 and **p < 0.01 vs. CUMS group.

To determine whether synaptic plasticity altered in the hippocampus of mice underwent chronic stress, we measured the synaptic protein expression of Synapsin-1 and PSD-95, and performed Golgi staining to observe dendritic spines (**Figure 4**). The protein levels of Synapsin-1 and PSD-95, as well as spine density in CUMS mice showed significant reduction compared with those in control group. While repeated administration of Cy and Flu evidently inhibited these reductions, suggesting that Cy and Flu were capable of maintaining the homeostasis of synaptic plasticity in CUMS mice.

### Pharmacological Inhibition of SIRT3 Impaired Cy-Induced Effects in CUMS Mice

#### SIRT3 Inhibitor Blocked Cy-Induced Rescue of Depressive Behaviors

**Figure 5:**
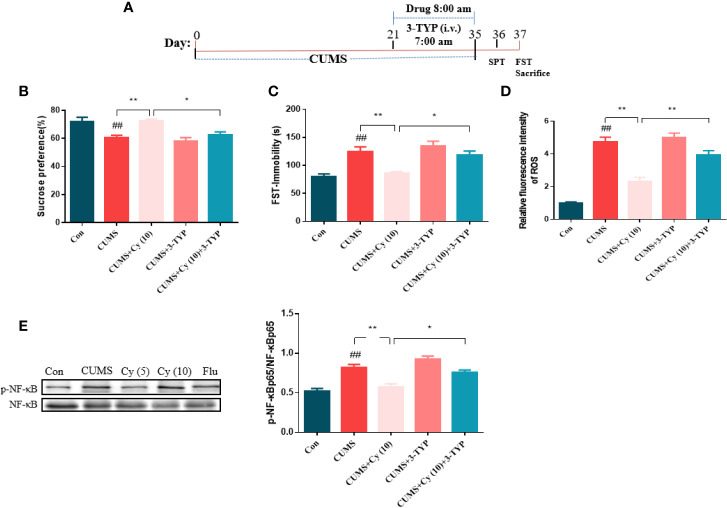
Effect of SIRT3 blockage on Cy-related attenuation on depressive behaviors, ROS production and NF-κB expression. 3-TYP was applied to inhibit SIRT3 signal **(A)**. The protective efficacy of Cy on SPT **(B)**, FST **(C)**, ROS production **(D)**, and NF-κB expression **(E)** were all reversed when co-administrated with SIRT3 inhibitor 3-TYP. Data are represented as mean ± SEM. ^##^*p* < 0.01 vs. Con group. *p < 0.05 and **p < 0.01 vs. CUMS + Cy (10 mg/kg) group.

To further investigate the functional role of SIRT3 in Cy-induced antidepressant efficacy, the SIRT3 inhibitor 3-TYP was used in the study (**Figure 5A**). Results from SPT and FST revealed that 3-TYP significantly blocked the antidepressive effects of Cy in SPT and FST (**Figures 5B, C**). These findings indicated that SIRT3 signaling was essential for the antidepressant effects of Cy in CUMS model.

#### SIRT3 Inhibitor Blocked Cy-Induced Restoration of ROS Production and NF-κB Expression

Next, we studied the impact of SIRT3 inhibitor on Cy-related attenuation of ROS production and NF-κB expression in CUMS mice. As illustrated in **Figures 5D, E**, the relative fluorescence intensity of ROS and phosphorylation rate of NF-κB were higher in the hippocampus of CUMS-treated group compared with control group, while Cy treatment significantly reduced ROS generation and NF-κB phosphorylation rate induced by CUMS. It is noteworthy that 3-TYP significantly reversed Cy administration induced decrease in NF-κB and ROS generation, indicating that Cy suppressed hippocampal ROS and NF-κB activities by activating SIRT3.

#### SIRT3 Blocked Cy-Induced Attenuation of NLRP3 Inflammasome and Neuroplasticity Deficits

**Figure 6:**
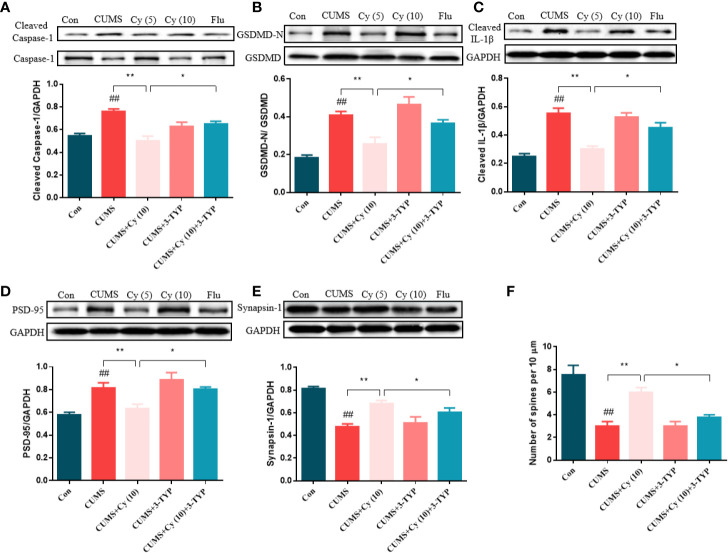
Effect of SIRT3 blockage on Cy-related improvements of NLRP3 inflammasome and neuroplasticity. Cy generated restoration of NLRP3 inflammasome proteins Caspase-1 **(A)**, GSDMD **(B)**, IL-1β **(C)**, and enhancement of PSD-95 **(D)**, Synapsin-1 expression **(E)** as well as dendritic spine density **(F)** were all abolished when co-administrated with SIRT3 inhibitor 3-TYP. Data are represented as mean ± SEM. ^##^*p* < 0.01 vs. Con group. *p < 0.05 and **p < 0.01 vs. CUMS + Cy (10 mg/kg) group.

To evaluate the impact of 3-TYP on Cy-generated effect on against NLRP3 inflammasome and synaptic plasticity, we examined protein levels related to NLRP3 inflammasome and synaptic plasticity, and performed Golgi staining to measure dendritic spine density. As the results shown in **Figure 6**, Cy significantly inhibited CUMS-induced activation of NLRP3 inflammasome in terms of Caspase-1, GSDMD and IL-1β. Moreover, the protein expressions of PSD-95 and Synapsin-1 and dendritic spine density was enhanced following Cy treatment. However, co-treatment with 3-TYP evidently reversed Cy-generated effects on NLRP3 inflammasome and neuroplasticity. These findings reflected that Cy-induced protective effects on NLRP3 inflammasome and synaptic plasticity were dependent on SIRT3 activation.

## Discussion

In the present study, we demonstrated that α-Cyperone from Cyperus rotundus exhibited potent antidepressant-like activities in CUMS mice, which was due to the neuroplasticity enhancement *via* SIRT3/ROS pathway mediated NLRP3 inflammasome deactivation. Furthermore, SIRT3 ablation abolished the therapeutic effects of Cy in CUMS mice.

CUMS is a classic model of depression since chronic stressful life events are vital cause of depression. In this model, rodents underwent CUMS challenge developed significant depressive disorders, including a marked decrease in response to rewards the clinical core symptom of depression (Willner, 2017). Fluoxetine, which served as the positive control in this study, is a widely used antidepressant that has been shown to exert distinguished antidepressive activity through the NLRP3 inflammation deactivation (Du et al., 2016; Alcocer-Gomez et al., 2017). In our study, anhedonia state and behavioral despair were detected in mice experienced CUMS, evidenced by decreased sucrose preference rate in SPT and increased immobility duration in TST and FST, suggesting the successful establishment of depression model. While, similar to Fluoxetine, Cy intervention notably mitigated these depressive symptoms, indicating that Cy possesses potent antidepressant efficacy.

In recent years, neuroplasticity, a feature of the brain’s response to internal and external stimuli, has been implicated in various neuropsychiatric disorders such as schizophrenia and bipolar disorder. Significant spine loss and alternations in dendritic spine density have been found in patients with schizophrenia disease (MacDonald et al., 2017). Lithium, the maintenance therapy used for the treatment of bipolar disorder, induced various positive effects for neuroplasticity, for instance, the enhancement of cellular proliferation, differentiation, and maturation (Machado-Vieira, 2018).

Neuroplasticity has also been involved in the progress of depression. Postmortem studies revealed smaller size of pyramidal neurons in the dorsolateral prefrontal cortex of depressed subjects relative to those of controls (Uchida et al., 2018). AC-5216, a selective TSPO ligand, manifested fast-onset antidepressant-like actions in chronically stressed mice, which was closely related to neuroplasticity improvement, including the upregulation of synaptic related proteins and restoration of dendritic structure (Shang et al., 2020). The ablation of neurogenesis in mice resulted in behavioral and biological changes relevant to depression. In line with these studies, our work showed that CUMS challenge decreased the protein levels of postsynaptic protein PSD-95 and presynaptic protein Synapsin-1, and reduced the dendritic spine density in the hippocampus of mice. However, Cy treatment enhanced PSD-95 and Synapsin-1 protein levels as well as dendritic spine density, reflecting the involvement of neuroplasticity in the etiology of depression and Cy-related amelioration on depressive symptoms. The NLRP3 inflammasome is a multi-protein signaling complex that can trigger the cleavage of Caspase-1, which facilitates the maturation of IL-1β and IL-18 into active cytokines and activates pyroptosis *via* GSDMD cleavage (Jo et al., 2016). The NLRP3 inflammasome has been proposed as a regulator of neuroplasticity. The declines in protein expression of NLRP3, ASC, Caspase-1, and IL-1βincreased BDNF and doublecortin (DCX) expressions, as well as BrdU+ cells in the hippocampuses of mice (Zuo et al., 2018). Spatial training reduced the production of NLRP3, Caspase-1, and IL-1β in PR5 mice, which upregulated the expression of synaptophysin, PSD-93, and PSD-95, and enhanced dendritic spine number (Ren et al., 2019).

The correlation between NLRP3 inflammasome and depression has also been documented. Clinical data suggested MDD patients demonstrated elevated NLRP3 and Caspase-1 mRNA expression in the peripheral blood mononuclear cells and increased IL-1β and IL-18 levels in the serum; serum IL-1β and IL-18 concentrations were positively related to the Beck Depression Inventory (BDI) scores of MDD patients (Alcocer-Gomez et al., 2014). Classical antidepressants such as paroxetine, amitriptyline and agomelatine were reported to reduce NLRP3, IL-1β, and IL-18 levels in patients with MDD (Alcocer-Gomez et al., 2017). Chronic stress promoted the production of NLRP3 inflammasome contents and inflammatory cytokine IL-1β and IL-18 (Wang et al., 2020). Compared with wild-type mice, NLRP3 gene knockout mice didn’t exhibit depression-like behaviors in SPT or TST after 4 weeks of CUMS exposure; meanwhile, NLRP3 gene knockout prevented the promotion of IL-1β in serum and hippocampi of CUMS mice (Su et al., 2017). Melatonin, a hormone produced from L-tryptophan mainly in the pineal gland, significantly reversed the depression-like behaviors induced by LPS, which was tightly linked with NLRP3 inflammasome inhibition generated decrease in GSDMD cleavage and pyroptotic cell death (Arioz et al., 2019). In this study, Cy effectively decreased the expression of NLRP3 inflammasome complex and cleaved IL-1β, IL-18, and GSDMD in mice received CUMS modeling, suggesting that Cy-related improvements in depressive behaviors and neuroplasticity defects were partially resulted from the suppression of NLRP3 inflammasome.

SIRT3, the most abundant sirtuin in the brain, is the only deacetylase in mitochondria that has robust deacetylase activity (Pillai et al., 2017). SIRT3 could deacetylate metabolic and respiratory enzymes that are correlated with electron transport chain function, consequently reduce the level of ROS, a key player in the mediation of NLRP3 inflammasome activity (Zheng et al., 2018). The SIRT3/ROS pathway is associated with the mediation of NLRP3 inflammasome. ROS stimulation dramatically promoted NLRP3 inflammasome generation, whereas the deletion of ROS significantly reduced the transcriptional levels of NLRP3, ASC, Caspase-1 and IL-1β (Dai et al., 2017). Qian Lei et al. studied the relationship between ROS and NLRP3-mediated pyroptosis on oxygen-glucose deprivation induced pyroptosis model. Their results showed that NLRP3 inflammasome-mediated pyroptosis could be prevented by the suppression of ROS, which hindered the phosphorylation of NF-κB and sequentially decreased the mRNA level of GSDMD and lactate dehydrogenase release (Lei et al., 2018).

The SIRT3/ROS pathway is also implicated in depression. The overproduction of ROS was detected in patients with depression (Bilici et al., 2001). The study conducted by Yu X et al. demonstrated that mice experienced chronic mild stress displayed deficiency in SIRT3 expression, as well as the overexpression of ROS, p-NF-κB p65 and IL-1β (Yu et al., 2020). Additionally, AICA Riboside intervention which restored hippocampal SIRT3 expression significantly ameliorated the depression-like and anxiety-like behaviors of CUMS mice. Nevertheless, the above protective effects of AICA Riboside were abolished when co-treated with SIRT3 antagonist 3-TYP. Consistent with these studies, our results showed declined protein level of SIRT3, and elevated ROS production and NF-κB phosphorylation rate in the hippocampus of CUMS mice, confirming the involvement of abnormal SIRT3/ROS/NF-κB signaling in the pathology of depression. However, Cy treatment significantly restored the SIRT3/ROS/NF-κB pathway, and inhibition of SIRT3 led to the ablation of Cy generated protective efficacy in CUMS mice, suggesting the crucial role of SIRT3 signaling in the treatment of depression.

**Figure 7:**
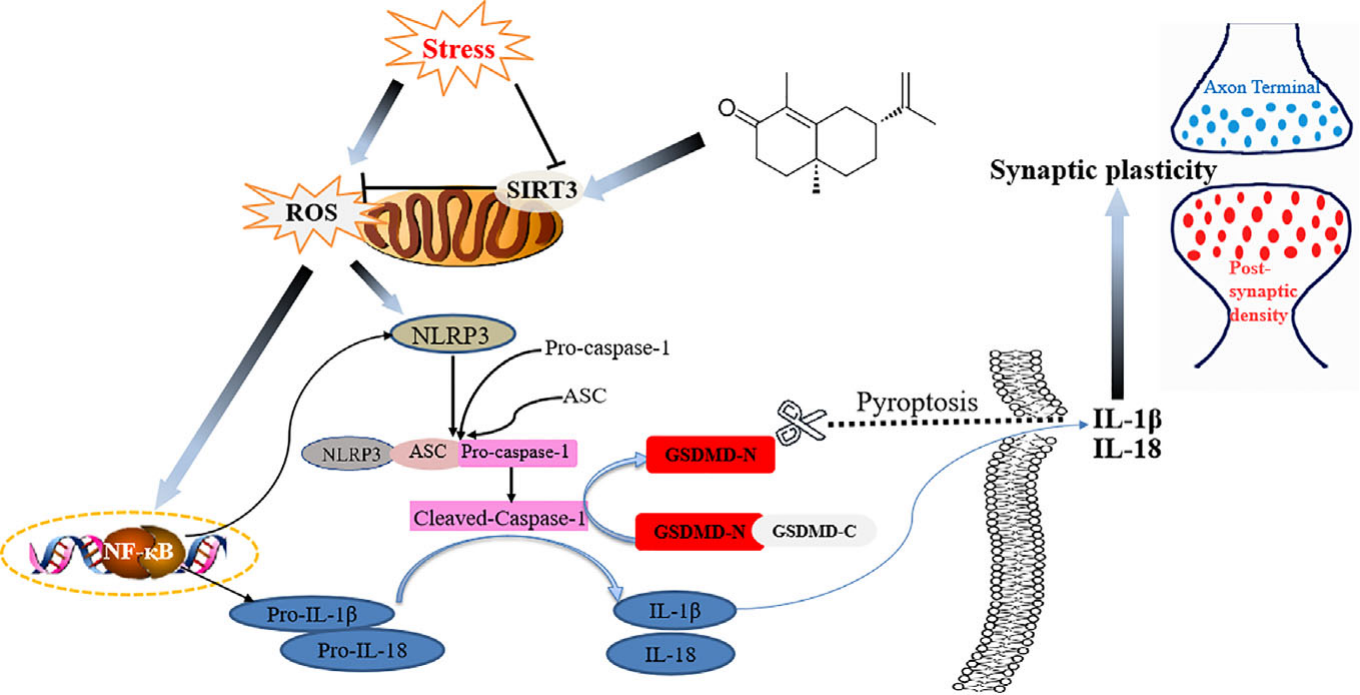
Schematic illustration of the proposed mechanism for Cy to ameliorate depression.

To conclude, for the first time to our knowledge, we demonstrated that α-Cyperone from Cyperus rotundus exerted antidepressant-like actions in a mouse depression model. The antidepressant activity of α-Cyperone was attributed to SIRT3/ROS pathway mediated NLRP3 inflammasome deactivation, which led to the enhancement of neuroplasticity (**Figure 7**). The findings revealed the antidepressant property of α-Cyperone, and provided support for targeting SIRT3/ROS signaling in depression treatment.

## Data Availability Statement

The raw data supporting the conclusions of this article will be made available by the authors, without undue reservation.

## Ethics Statement

The animal study was reviewed and approved by Animal Ethics Committee of Nanjing University of Chinese Medicine.

## Author Contributions

BX and XS provided financial support for the conduct of the research and preparation of the article. BX, YT, and XS wrote the main manuscript text. BX, YT, and CX prepared **Figures 1**–**4**. BX, YT, CC, and XS prepared **Figures 5**–**7**. All authors contributed to the article and approved the submitted version.

## Funding

This work was supported by the National Natural Science Foundation of China (81804068), Natural Science Foundation of Jiangsu Province (BK20170769), Natural science fund for colleges and universities in Jiangsu Province (17KJD360001), Blue Project of Jiangsu Province and Overseas study program for excellent young and middle-aged teachers and principals of universities in Jiangsu province., Natural Science Foundation of colleges and universities in Jiangsu (NO.18KJB360011); Jiangsu TCM science and technology development program (NO.YB201997). Postgraduate Research & Practice Innovation Program of Jiangsu Province (NO.KYCX20_1496).

## Conflict of Interest

The authors declare that the research was conducted in the absence of any commercial or financial relationships that could be construed as a potential conflict of interest.

## References

[B1] Alcocer-Gomez E. Casas-Barquero N. Williams M. R. Romero-Guillena S. L. Canadas-Lozano D. Bullon P. (2017). Antidepressants induce autophagy dependent-NLRP3-inflammasome inhibition in Major depressive disorder. Pharmacol. Res. 121, 114–121. 10.1016/j.phrs.2017.04.028 28465217

[B2] Alcocer-Gomez E. de Miguel M. Casas-Barquero N. Nunez-Vasco J. Sanchez-Alcazar J. A. Fernandez-Rodriguez A. (2014). NLRP3 inflammasome is activated in mononuclear blood cells from patients with major depressive disorder. Brain Behav. Immun. 36, 111–117. 10.1016/j.bbi.2013.10.017 24513871

[B3] Arioz B. I. Tastan B. Tarakcioglu E. Tufekci K. U. Olcum M. Ersoy N. (2019). Melatonin Attenuates LPS-Induced Acute Depressive-Like Behaviors and Microglial NLRP3 Inflammasome Activation Through the SIRT1/Nrf2 Pathway. Front. Immunol. 10, 1511. 10.3389/fimmu.2019.01511 31327964 PMC6615259

[B4] Arroyo-Garcia L. E. Tendilla-Beltran H. Vazquez-Roque R. A. Jurado-Tapia E. E. Diaz A. Aguilar-Alonso P. (2020). Amphetamine sensitization alters hippocampal neuronal morphology and memory and learning behaviors. Mol. Psychiatry 2020, 1–11. 10.1038/s41380-020-0809-2 32555421

[B5] Azimi A. Ghaffari S. M. Riazi G. H. Arab S. S. Tavakol M. M. Pooyan S. (2016). alpha-Cyperone of Cyperus rotundus is an effective candidate for reduction of inflammation by destabilization of microtubule fibers in brain. J. Ethnopharmacol. 194, 219–227. 10.1016/j.jep.2016.06.058 27353867

[B6] Bilici M. Efe H. Koroglu M. A. Uydu H. A. Bekaroglu M. Deger O. (2001). Antioxidative enzyme activities and lipid peroxidation in major depression: alterations by antidepressant treatments. J. Affect. Disord. 64 (1), 43–51. 10.1016/s0165-0327(00)00199-3 11292519

[B7] Clayton A. H. Croft H. A. Yuan J. Brown L. Kissling R. (2018). Safety of Flibanserin in Women Treated With Antidepressants: A Randomized, Placebo-Controlled Study. J. Sex Med. 15 (1), 43–51. 10.1016/j.jsxm.2017.11.005 29289374

[B8] Dai J. Zhang X. Li L. Chen H. Chai Y. (2017). Autophagy Inhibition Contributes to ROS-Producing NLRP3-Dependent Inflammasome Activation and Cytokine Secretion in High Glucose-Induced Macrophages. Cell Physiol. Biochem. 43 (1), 247–256. 10.1159/000480367 28854426

[B9] Du R. H. Tan J. Sun X. Y. Lu M. Ding J. H. Hu G. (2016). Fluoxetine Inhibits NLRP3 Inflammasome Activation: Implication in Depression. Int. J. Neuropsychopharmacol. 19 (9), 1–9. 10.1093/ijnp/pyw037 PMC5043644 27207922

[B10] Feng X. Zhao Y. Yang T. Song M. Wang C. Yao Y. (2019). Glucocorticoid-Driven NLRP3 Inflammasome Activation in Hippocampal Microglia Mediates Chronic Stress-Induced Depressive-Like Behaviors. Front. Mol. Neurosci. 12, 210. 10.3389/fnmol.2019.00210 31555091 PMC6727781

[B11] Gentile D. Fornai M. Pellegrini C. Colucci R. Benvenuti L. Duranti E. (2018). Luteolin Prevents Cardiometabolic Alterations and Vascular Dysfunction in Mice With HFD-Induced Obesity. Front. Pharmacol. 9, 1094. 10.3389/fphar.2018.01094 30319424 PMC6167518

[B12] Jo E. K. Kim J. K. Shin D. M. Sasakawa C. (2016). Molecular mechanisms regulating NLRP3 inflammasome activation. Cell Mol. Immunol. 13 (2), 148–159. 10.1038/cmi.2015.95 26549800 PMC4786634

[B13] Jung S. H. Kim S. J. Jun B. G. Lee K. T. Hong S. P. Oh M. S. (2013). alpha-Cyperone, isolated from the rhizomes of Cyperus rotundus, inhibits LPS-induced COX-2 expression and PGE2 production through the negative regulation of NFkappaB signalling in RAW 264.7 cells. J. Ethnopharmacol. 147 (1), 208–214. 10.1016/j.jep.2013.02.034 23500883

[B14] Laura G. Silvia T. Nikolaos P. Patrizia P. (2018). The Role of fMRI in the Assessment of Neuroplasticity in MS: A Systematic Review. Neural Plast. 2018, 3419871. 10.1155/2018/3419871 30693023 PMC6332922

[B15] Lei Q. Yi T. Chen C. (2018). NF-kappaB-Gasdermin D (GSDMD) Axis Couples Oxidative Stress and NACHT, LRR and PYD Domains-Containing Protein 3 (NLRP3) Inflammasome-Mediated Cardiomyocyte Pyroptosis Following Myocardial Infarction. Med. Sci. Monit. 24, 6044–6052. 10.12659/MSM.908529 30161099 PMC6128186

[B16] Liu X. Jin X. Yu D. Liu G. (2019). Suppression of NLRP3 and NF-kappaB signaling pathways by alpha-Cyperone via activating SIRT1 contributes to attenuation of LPS-induced acute lung injury in mice. Int. Immunopharmacol. 76, 105886. 10.1016/j.intimp.2019.105886 31520990

[B17] MacDonald M. L. Alhassan J. Newman J. T. Richard M. Gu H. Kelly R. M. (2017). Selective Loss of Smaller Spines in Schizophrenia. Am. J. Psychiatry 174 (6), 586–594. 10.1176/appi.ajp.2017.16070814 28359200 PMC5800878

[B18] Machado-Vieira R. (2018). Lithium, Stress, and Resilience in Bipolar Disorder: Deciphering this key homeostatic synaptic plasticity regulator. J. Affect. Disord. 233, 92–99. 10.1016/j.jad.2017.12.026 29310970

[B19] McAvoy K. Russo C. Kim S. Rankin G. Sahay A. (2015). Fluoxetine induces input-specific hippocampal dendritic spine remodeling along the septotemporal axis in adulthood and middle age. Hippocampus 25 (11), 1429–1446. 10.1002/hipo.22464 25850664 PMC4596739

[B20] Menard C. Hodes G. E. Russo S. J. (2016). Pathogenesis of depression: Insights from human and rodent studies. Neuroscience 321, 138–162. 10.1016/j.neuroscience.2015.05.053 26037806 PMC4664582

[B21] Pillai V. B. Kanwal A. Fang Y. H. Sharp W. W. Samant S. Arbiser J. (2017). Honokiol, an activator of Sirtuin-3 (SIRT3) preserves mitochondria and protects the heart from doxorubicin-induced cardiomyopathy in mice. Oncotarget 8 (21), 34082–34098. 10.18632/oncotarget.16133 28423723 PMC5470953

[B22] Qi Y. Klyubin I. Cuello A. C. Rowan M. J. (2018). NLRP3-dependent synaptic plasticity deficit in an Alzheimer’s disease amyloidosis model in vivo. Neurobiol. Dis. 114, 24–30. 10.1016/j.nbd.2018.02.016 29477641

[B23] Ren Q. G. Gong W. G. Zhou H. Shu H. Wang Y. J. Zhang Z. J. (2019). Spatial Training Ameliorates Long-Term Alzheimer’s Disease-Like Pathological Deficits by Reducing NLRP3 Inflammasomes in PR5 Mice. Neurotherapeutics 16 (2), 450–464. 10.1007/s13311-018-00698-w 30560481 PMC6554388

[B24] Shang C. Yao R. M. Guo Y. Ding Z. C. Sun L. J. Ran Y. H. (2020). Translocator protein-mediated fast-onset antidepressant-like and memory-enhancing effects in chronically stressed mice. J. Psychopharmacol. 34 (4), 441–451. 10.1177/0269881119896304 31913078

[B25] Shao B. Z. Cao Q. Liu C. (2018). Targeting NLRP3 Inflammasome in the Treatment of CNS Diseases. Front. Mol. Neurosci. 11, 320. 10.3389/fnmol.2018.00320 30233319 PMC6131647

[B26] Su W. J. Zhang Y. Chen Y. Gong H. Lian Y. J. Peng W. (2017). NLRP3 gene knockout blocks NF-kappaB and MAPK signaling pathway in CUMS-induced depression mouse model. Behav. Brain Res. 322 (Pt A), 1–8. 10.1016/j.bbr.2017.01.018 28093255

[B27] Teng J. F. Mei Q. B. Zhou X. G. Tang Y. Xiong R. Qiu W. Q. (2020). Polyphyllin VI Induces Caspase-1-Mediated Pyroptosis via the Induction of ROS/NF-kappaB/NLRP3/GSDMD Signal Axis in Non-Small Cell Lung Cancer. Cancers (Basel) 12 (1), 193. 10.3390/cancers12010193 PMC7017302 31941010

[B28] Uchida S. Yamagata H. Seki T. Watanabe Y. (2018). Epigenetic mechanisms of major depression: Targeting neuronal plasticity. Psychiatry Clin. Neurosci. 72 (4), 212–227. 10.1111/pcn.12621 29154458

[B29] Wang D. Wang H. Gao H. Zhang H. Zhang H. Wang Q. (2020). P2X7 receptor mediates NLRP3 inflammasome activation in depression and diabetes. Cell Biosci. 10, 28. 10.1186/s13578-020-00388-1 32166013 PMC7059335

[B30] Wang G. Lei C. Tian Y. Wang Y. Zhang L. Zhang R. (2019). Rb1, the Primary Active Ingredient in Panax ginseng C.A. Meyer, Exerts Antidepressant-Like Effects via the BDNF-Trkb-CREB Pathway. Front. Pharmacol. 10, 1034. 10.3389/fphar.2019.01034 31572200 PMC6753202

[B31] Willner P. (2017). The chronic mild stress (CMS) model of depression: History, evaluation and usage. Neurobiol. Stress 6, 78–93. 10.1016/j.ynstr.2016.08.002 28229111 PMC5314424

[B32] Yu X. Hu Y. Huang W. Ye N. Yan Q. Ni W. (2020). Role of AMPK/SIRT1-SIRT3 signaling pathway in affective disorders in unpredictable chronic mild stress mice. Neuropharmacology 165, 107925. 10.1016/j.neuropharm.2019.107925 31877320

[B33] Zheng J. Shi L. Liang F. Xu W. Li T. Gao L. (2018). Sirt3 Ameliorates Oxidative Stress and Mitochondrial Dysfunction After Intracerebral Hemorrhage in Diabetic Rats. Front. Neurosci. 12, 414. 10.3389/fnins.2018.00414 29970985 PMC6018086

[B34] Zuo Y. Wang J. Liao F. Yan X. Li J. Huang L. (2018). Inhibition of Heat Shock Protein 90 by 17-AAG Reduces Inflammation via P2X7 Receptor/NLRP3 Inflammasome Pathway and Increases Neurogenesis After Subarachnoid Hemorrhage in Mice. Front. Mol. Neurosci. 11, 401. 10.3389/fnmol.2018.00401 30459553 PMC6232389

